# Novel Role of GPR35 (G-Protein–Coupled Receptor 35) in the Regulation of Endothelial Cell Function and Blood Pressure

**DOI:** 10.1161/HYPERTENSIONAHA.120.15423

**Published:** 2021-07-19

**Authors:** Hainan Li, Huong Nguyen, Sai Pranathi Meda Venkata, Jia Yi Koh, Anjaneyulu Kowluru, Li Li, Noreen F. Rossi, Wei Chen, Jie-Mei Wang

**Affiliations:** Department of Pharmaceutical Sciences, Eugene Applebaum College of Pharmacy and Health Sciences; Department of Pharmaceutical Sciences, Eugene Applebaum College of Pharmacy and Health Sciences; Department of Pharmaceutical Sciences, Eugene Applebaum College of Pharmacy and Health Sciences; Department of Pharmaceutical Sciences, Eugene Applebaum College of Pharmacy and Health Sciences; Department of Pharmaceutical Sciences, Eugene Applebaum College of Pharmacy and Health Sciences; John D. Dingell VA Medical Center, Detroit, MI; Departments of Internal Medicine, Wayne State University, Detroit, MI; Departments of Internal Medicine, Wayne State University, Detroit, MI; John D. Dingell VA Medical Center, Detroit, MI; Department of Oncology, Wayne State University, Detroit, MI; School of Medicine, Karmanos Cancer Institute, Wayne State University, Detroit, MI; Department of Pharmaceutical Sciences, Eugene Applebaum College of Pharmacy and Health Sciences; Centers for Molecular Medicine and Genetics, Wayne State University, Detroit, MI

**Keywords:** blood pressure, cardiovascular diseases, GTP cyclohydrolase, homeostasis, nitric oxide

## Abstract

GPR35 (G-protein–coupled receptor 35) is a poorly characterized receptor that has garnered increased interest as a therapeutic target through its implications in a range of inflammatory and cardiovascular diseases, but its biological functions stay largely unknown. The current study evaluated the effect of GPR35 on endothelial cell (EC) functions and hemodynamic homeostasis. In primary human aortic ECs, the expression of GPR35 was manipulated by transfections of adenovirus carrying either GPR35 cDNA or shRNA against GPR35, using adenovirus carrying β-gal as control. Mouse aortic ECs were isolated and cultured from GPR35 knockout and wild-type control mice. Our results indicated that genetic inhibition of GPR35 in human and mouse ECs significantly promoted cell proliferation, migration, and tube formation in vitro. The GCH1 (guanosine triphosphate cyclohydrolase I)-mediated biosynthesis of tetrahydrobiopterin was enhanced, reducing intracellular superoxide. Knocking down GCH1 or eNOS (endothelial nitric oxide synthase) significantly blunted the robust angiogenesis induced by GPR35 suppression. Male GPR35 knockout mice demonstrated reduced basal arterial blood pressure and an attenuated onset of hypertension in deoxycorticosterone acetate-salt induced hypertensive model compared with male GPR35 wild-type control mice in vivo, with concomitant improved endothelium-dependent vasodilation and decreased superoxide in isolated aortas. The difference in arterial blood pressure was absent between female GPR35 wild-type control and female GPR35 knockout mice. Our study provides novel insights into the roles of GPR35 in endothelial function and vascular tone modulation that critically contribute to the pathophysiology of blood pressure elevation. Antagonizing GPR35 activity might represent a potentially effective therapeutic approach to restore EC function and hemodynamic homeostasis.

Elevated blood pressure (BP; elevated pressure, stage 1, or stage 2 hypertension) is a major risk factor for cardiovascular events.^1^ The endothelial dysfunction is a crucial feature of hypertension.^2,3^ Ample evidence has implicated a vicious cycle between hypertension and endothelial dysfunction,^4^ but the underlying mechanisms are not fully understood. Endothelium-derived nitric oxide (NO) is a potent vasodilator that maintains cardiovascular homeostasis.^5^ Patients with hypertension or animals possess blunted endothelium-dependent NO-mediated vasodilation,^4,6^ which is a valuable indicator for endothelial dysfunction at the early onset of hypertension.^7^ The activation of the eNOS (endothelial NO synthase) and the capacity of eNOS to generate NO are tightly controlled by multiple integrative signaling pathways.^8^ The deficiency of tetrahydrobiopterin (BH4), the cofactor of eNOS, results in eNOS uncoupling, which switches eNOS to generate superoxide rather than NO, leading to endothelial dysfunction and the progression of atherosclerosis.^9,10^ The rapid oxidation of BH4 makes it challenging for therapeutic use through oral and intravenous administration.^11–13^ Alternatively, overexpression of GCH1 (guanosine triphosphate cyclohydrolase I), the rate-limiting enzyme in the biosynthesis of BH4,^14^ has demonstrated excellent curative effects in animal models of hypertension,^15,16^ but the method is not ready for clinical use. Therefore, there is a compelling need to search for druggable targets that can modulate GCH1-mediated BH4 biosynthesis.

GPCRs (G-protein–coupled receptors; or GPRs) are a family of 7-transmembrane proteins representing the largest group of druggable targets.^17^ However, the physiological functions of many GPRs are still unknown. GPR35 is a recently deorphanized GPCR that has been suggested as a novel therapeutic target based on the association between GPR35 polymorphisms and various pathologies,^18,19^ including heart failure,^20–23^ diabetes,^24^ inflammatory bowel disease,^25^ and cancer.^26^ We reported that GPR35 participated in experimental colitis by modulating the inflammatory response.^27^ However, the precise function of GPR35 in the cardiovascular system remains to be elucidated. A previous report showed an increased BP profile in GPR35 knockout mice under anesthesia compared with the wild-type controls.^20^ Yet in another study, mice with GPR35 deletion showed resistance to BP elevation in Ang II (angiotensin II)–induced hypertension.^22^ To date, the potential mechanisms of GPR35 actions in endothelial function and BP regulation are not clear.

In this study, we tested the effect of genetic inhibition of GPR35 on angiogenesis and eNOS activation in mouse and human aortic endothelial cells (ECs) in vitro. We also tested whether GPR35 regulated endothelium-dependent vasodilation in isolated vessels. We explored the possible pathways through which the GPR35 attenuated eNOS activation and induced oxidative stress in ECs. Furthermore, whether GCH1 plays an essential role in GPR35-regulated angiogenesis was tested. The in vivo BP profile of male and female GPR35 knockout mice and their wild-type controls in deoxycorticosterone acetate (DOCA)-salt induced hypertension was acquired.

## METHODS

The data that support the findings of this study are available from the corresponding author upon reasonable request. An expanded Material and Methods section is available in the Data Supplement.

### Animal Procedures

GPR35 knockout mice (GPR35^KO^, on the C57BL/6 background) were provided by Wellcome Trust Sanger Institute (Cambridge, England).^28,29^ GPR35^KO^ mice were bred with C57BL/6 mice (purchased from Jackson Laboratory, Bar Harbor, ME) to generate GPR35^KO^ and their wild-type control (GPR35^WT^). According to the National Institutes Guide for the Care and Use of Laboratory Animals, all animal experiments were performed and approved by the Wayne State University Institutional Animal Care and Use Committee.

### Telemetry Transmitter Implantation and Data Acquisition

A telemetry transmitter (Data Sciences International, Model TA11PA-C10) was implanted in the left carotid artery of the animal under isoflurane anesthesia. After the animal recovered, continuous BP was acquired using Ponemah software (Data Sciences International).

### DOCA-Salt Induced Hypertensive Mouse Model

The systolic, diastolic, mean arterial BP, and heart rate of the mice were measured for 5 consecutive days by the noninvasive tail-cuff method (Kent Scientific). The mice in DOCA-salt group had their left kidneys removed. The mice were implanted subcutaneously with a DOCA pellet (Innovative Research of America) and provided with drinking water containing 1.0% NaCl and 0.2% KCl. The mice in the sham group only had their left kidney removed and were given tap water. Mice in N^G^-nitro-L-arginine methyl ester hydrochloride (L-NAME)-treated groups were administered L-NAME (Sigma) in drinking water for 4 weeks after the surgery.

### Vasorelaxation Assay

The vasorelaxation assay was performed using an in vitro wire myography system (Wire Myograph 620M, Danish Myo Technology, Denmark). Aortic rings (3 mm) were preconstricted at 3 millinewtons (mN) and maintained in physiological saline solution (aerated with 95% O_2_/5% CO_2_). The vessels were then reactivated by incubation with high potassium buffer. Then the vessels were constricted with Phenylephrine. After a steady-state was achieved, the cumulative concentrations of acetylcholine (10^−9^ to 10^−4^ mol/L) were added to induce relaxant responses.

### Western Blot Analysis

The cell lysate was subjected to Western blot analysis using primary antibodies directed against each target molecule: eNOS (no. 32027), phospho-eNOS (p-eNOS, no. 9571), GFP (green fluorescent protein; no. 2956), β-actin (no. 12262) from Cell Signaling, GCH1 (NBP1–79771) from NOVUS, AT1 (sc-515884) from Santa Cruz. After incubation with secondary antibodies, the blot was read with a C-Digit imager (Li-Cor). Quantitative analysis of protein levels was analyzed with Image Studio Lite Ver 5.2 (Li-Cor).

### Statistical Analysis

All values are expressed as mean±SD. For continuous variables that failed Shiparo-Wilk normality tests, such as mRNA expression, protein levels, staining quantifications, functional assays, the statistical significance of differences between the 2 groups was determined with the Mann-Whitney *U* test. In myograph data, the 2 groups were tested using multiple Mann-Whitney *U* tests with Benjamini Krieger, and Yekutieli’s adjustment.^30^ When >2 groups of treatments were performed, we used the Kruskal-Wallis test across all the groups, and if significant, we then tested the pairs of our primary interest based on scientific rationale using the Mann-Whitney *U* test with Hommel’s adjustment for multiple comparisons.^31^ These gatekeeping approaches and the adjustments preserved alpha spending and controlled false positive rate inflation due to multiple hypothesis testing. The significant differences that came from post hoc comparisons of groups were noted. Similarly, for continuous variables that follow the normal distributions, such as BP and heart rates, the statistical significance of differences between the 2 groups was determined by the Student *t* test. When >2 treatment groups were performed, 1-way ANOVA was used across all the groups, and if significant, pairs of focused groups were tested with 2-sample *t* test and *P* values were adjusted with Hommel method. A value of *P*<0.05 was considered statistically significant. All the statistical analyses were performed using GraphPad Prism 9 (GraphPad Software). The Hommel’s adjustment was performed using R version 3.6.3.

## RESULTS

### The Deletion of GPR35 Enhances Angiogenesis In Vitro

**Figure 1.**
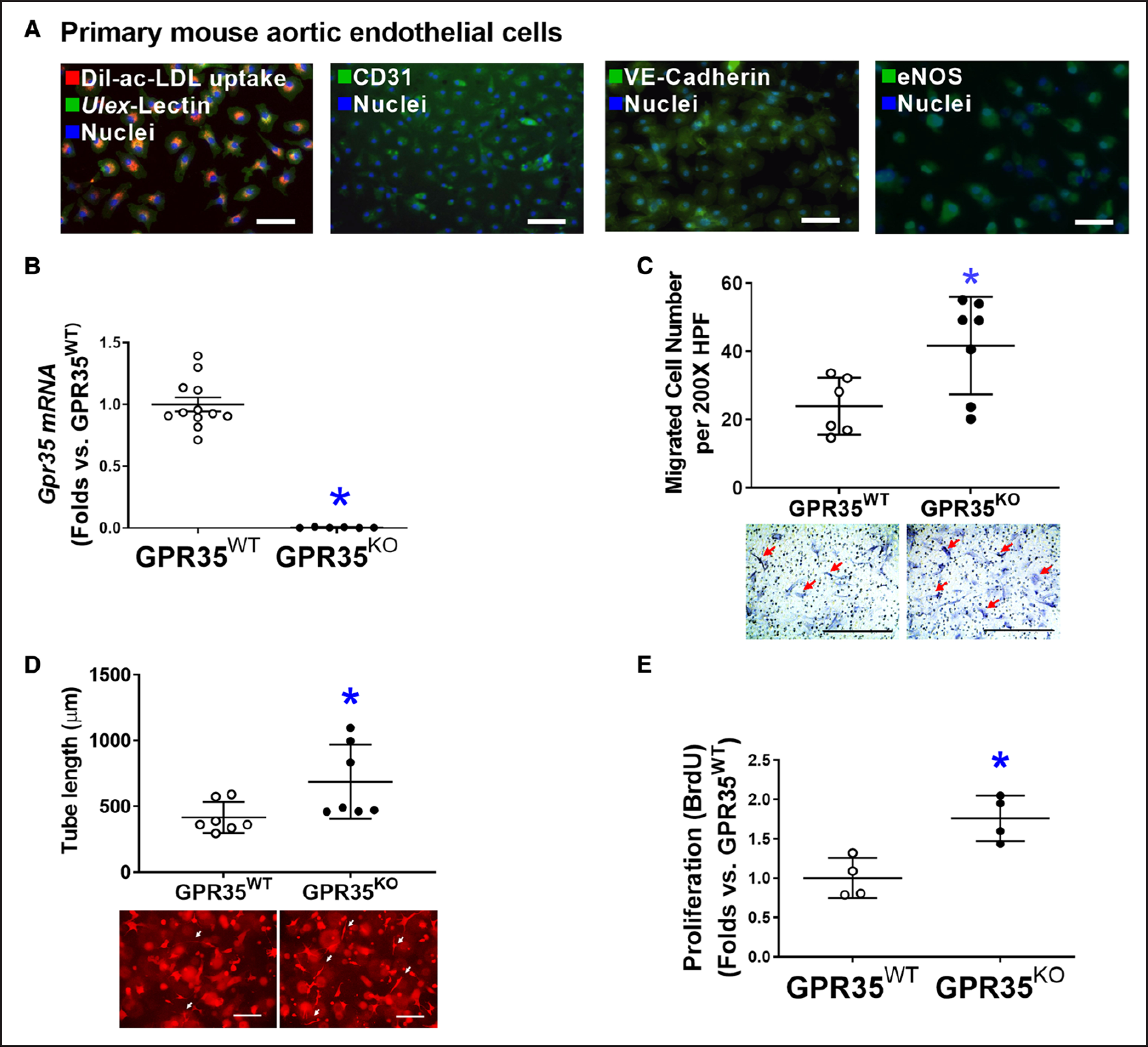
Genetic ablation of GPR35 (G-protein–coupled receptor 35) in aortic endothelial cells (ECs) improves cell functions. **A**, Representative fluorescent images showing characteristics of mouse aortic ECs (MAECs) by fluorescent staining of Dil-ac-LDL uptake and Ulex-lectin binding, CD31, VE-Cadherin, and eNOS (endothelial nitric oxide synthase). Scale bar, 100 μm. **B**, Real-time quantitative polymerase chain reaction (qPCR) confirmed the expression levels of *Gpr35* in the GPR35^WT^ and little to no expression in the GPR35^KO^ MAECs. n=6–12, *P<0.05. **C**, Cell migration assay in GPR35^WT^ and GPR35^KO^ MAECs. n=6 per group. Scale bar, 400 μm. **D**, Three-dimensional (3D) tube formation assay in GPR35^WT^ and GPR35^KO^ MAECs. n=7 group. Scale bar, 400 μm. **E**, Cell proliferation assay in GPR35^WT^ and GPR35^KO^ MAECs. n=4 group. In **B**–**E**, Mann-Whitney *U* tests were used, *P<0.05 vs GPR35^WT^ ECs, horizontal lines show mean±SD. DAPI indicates 4’,6-diamidino-2-phenylindole.

As shown in Figure 1A, primary cultured mouse aortic ECs (MAECs) possessed spindle-shaped and cobblestone-like appearances, double-positive for Dil-ac-LDL uptake and *Ulex*-lectin binding, positive for platelet endothelial cell adhesion molecule 1 (CD31), and expressing EC functional proteins such as VE-Cadherin and eNOS, all of which were characteristics of endothelial lineage. Quantitative real-time polymerase chain reactions verified the absence of GPR35 in GPR35^KO^ MAECs (Figure 1B). The functional assay results demonstrated that GPR35 deletion significantly promoted the migration (Figure 1C, n=6 in GPR35^WT^, n=7 in GPR35^KO^, Mann-Whitney *U* test *P*=0.035), 3-dimensional (3D) tube formation (Figure 1D, n=7 per group, Mann-Whitney *U* test *P*=0.038), and proliferation of MAECs (Figure 1E, n=4 per group, Mann-Whitney *U* test *P*=0.029).

**Figure 2.**
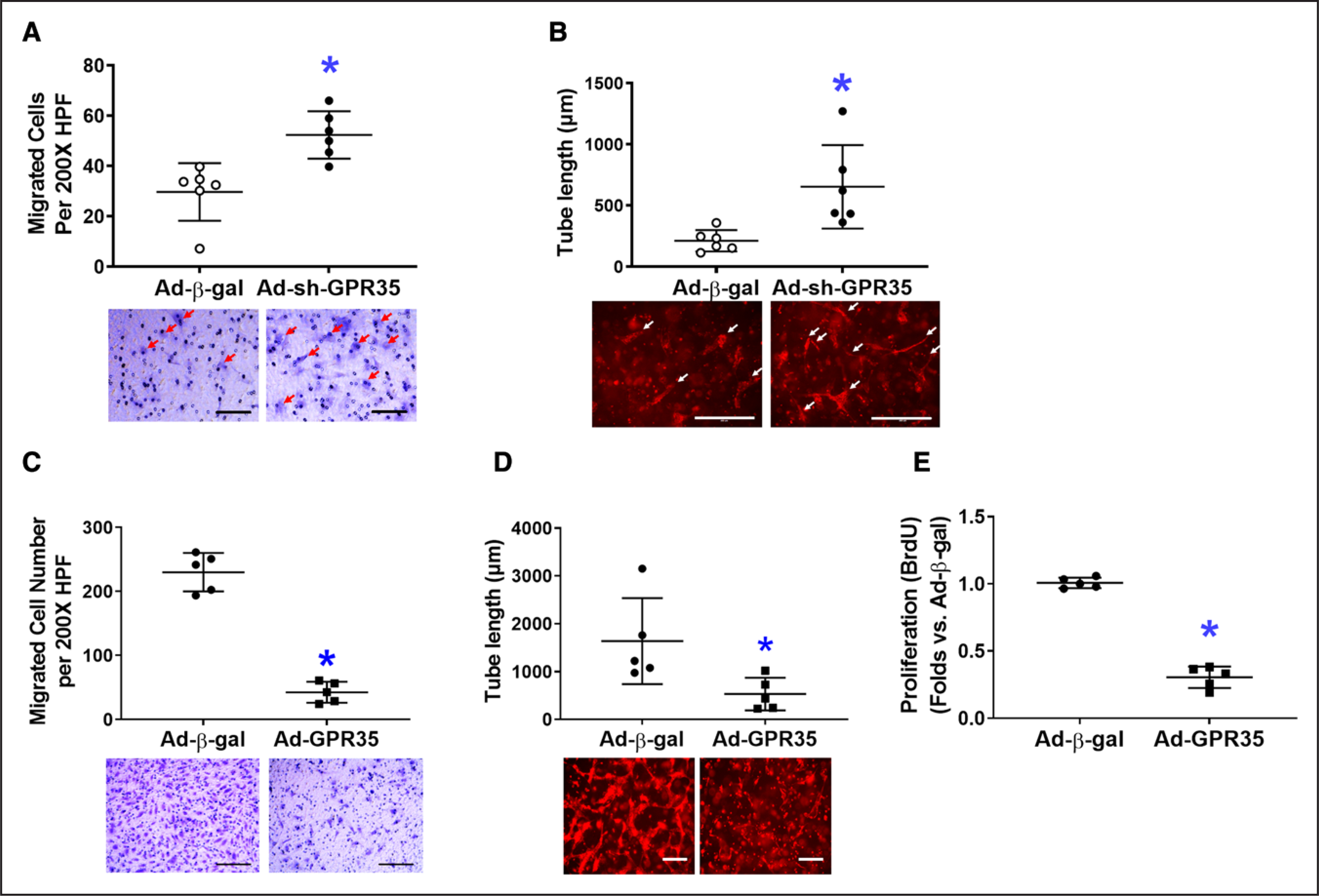
Genetic inhibition of GPR35 (G-protein–coupled receptor 35) increased nitric oxide (NO) production and reduced reactive oxygen species in human aortic endothelial cells (HAECs) while improving cell functions. In HAECs, the GPR35 expression was knocked down by the transfection of an adenovirus carrying shRNA against the human GPR35 gene (Ad-sh-GPR35), or overexpressed by an adenovirus carrying human GPR35 gene (Ad-GPR35), using adenovirus carrying β-gal (Ad-β-gal) as control. Representative images are shown underneath the dot-plot figure. Scale bar=200 μm. **A**, Cell migration assay in HAECs with Ad-sh-GPR35 or Ad-β-gal. n=6, *P<0.05 vs Ad-β-gal. **B**, Three-dimensional (3D) tube formation assay in HAECs with Ad-sh-GPR35 or Ad-β-gal. Scale bar=200 μm. n=6 per group, *P<0.05 vs Ad-β-gal. Scale bar=200 μm. n=6 per group, *P<0.05 vs Ad-β-gal. **C**, Cell migration assay in HAECs with Ad-GPR35 or Ad-β-gal. n=5 per group, *P<0.05 vs Ad-β-gal. **D**, 3D tube formation assay in HAECs with Ad-GPR35 or Ad-β-gal. n=5 per group, *P<0.05 vs Ad-β-gal. **E**, Cell proliferation assay in HAECs with Ad-GPR35 or Ad-β-gal. n=5 per group, *P<0.05 vs Ad-β-gal. In the figures, Mann-Whitney *U* tests were used, horizontal lines show mean±SD.

In primary human aortic ECs (HAECs), GPR35 was knocked down by a GFP-tagged adenoviral vector expressing shRNA against human GPR35 tagged (Ad-sh-GPR35, 50 MOI, 48 hours), using adenovirus carrying *β*-gal as a control. Western blot analysis demonstrated the successful transfection of GFP-tagged Ad-sh-GPR35 (Figure S1A in the Data Supplement). The quantitative real-time polymerase chain reaction data confirmed the suppression of GPR35 by Ad-sh-GPR35 in HAECs (Figure S1B). The functional assays suggested that knocking down GPR35 in HAECs significantly improved migration (Figure 2A, n=6 per group, Mann-Whitney *U* test *P*=0.004) and 3D tube formation (Figure 2B, n=6 per group, Mann-Whitney *U* test *P*=0.002). When GPR35 in HAECs was overexpressed by an adenoviral vector carrying human GPR35 (Ad-GPR35, mRNA elevation confirmed by quantitative real-time polymerase chain reaction in Figure S1C), the migration (Figure 2C, n=5 per group, Mann-Whitney *U* test *P*=0.008), 3D tube formation (Figure 2D, n=5 per group, Mann-Whitney *U* test *P*=0.016), and proliferation (Figure 2E, n=5 per group, Mann-Whitney *U* test *P*=0.008) were significantly impaired. These observations suggest that the inhibition of GPR35 in mouse and human ECs results in enhanced angiogenesis in vitro.

### GPR35 Attenuates eNOS Phosphorylation and Reduces NO Bioavailability

**Figure 3.**
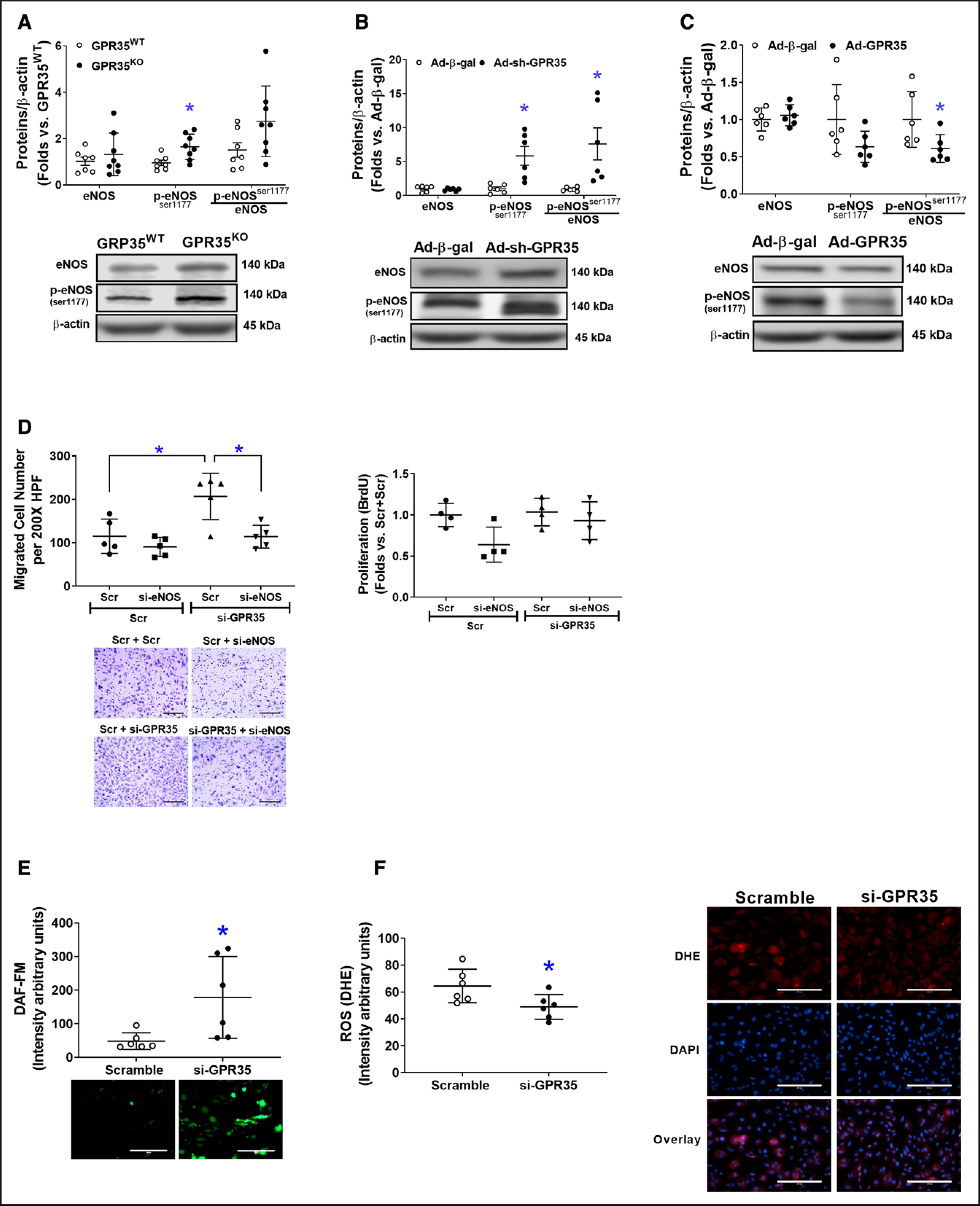
Deletion of GPR35 (G-protein–coupled receptor 35) increased eNOS (endothelial nitric oxide synthase) activation in human aortic endothelial cells (HAECs). The protein levels of total eNOS (noted as eNOS), phosphorylated eNOS at Ser1177(p-eNOS^ser1177^), and β-actin were determined by Western blot analysis using β-actin as a housekeeping protein. The extent of eNOS phosphorylation was calculated as the ratio of p-eNOS (Ser1177) to total eNOS (p-eNOS^ser1177^/eNOS). Representative images are shown beneath the quantifications. **A**, The eNOS, p-eNOS^ser1177^, and p-eNOS^ser1177^/eNOS in GPR35^WT^ and GPR35^KO^ mouse aortic ECs (MAECs). n=7 in GPR35^WT^, n=8 in GPR35^KO^ ECs. Mann-Whitney *U* tests were used. *P<0.05 vs GPR35^WT^. **B**, The eNOS, p-eNOS^ser1177^, and p-eNOS^ser1177^/eNOS in HAECs infected with Ad-sh-GPR35 or Ad-*β*-gal. n=6 per group. Mann-Whitney *U* tests were used. *P<0.05 vs Ad-Ad-*β*-gal. **C**, The eNOS, p-eNOS^ser1177^, and p-eNOS^ser1177^/eNOS in HAECs infected with Ad-sh-GPR35 or Ad-*β*-gal. n=6 per group. Mann-Whitney *U* tests were used. *P<0.05 vs Ad-Ad-*β*-gal. **D**, Functional assays including migration (**left**) and proliferation (**right**) in HAECs transfected with siRNA against eNOS (si-eNOS) and siRNA against GPR35 (si-GPR35), using scramble oligo as control. Representative images are shown next to the quantifications. Scale bar, 200 μm. n=5 per group in the migration assay, n=4 per group in the proliferation assay. Kruskal-Wallis tests were done, followed by Mann-Whitney *U* tests with Hommel’s adjustment. *P<0.05 between the 2 groups in post hoc comparisons. **E**, Quantification of fluorescent staining of NO production in HAECs transfected with siRNA against GPR35 (si-GPR35), using scramble oligo as control. Representative images are shown. Scale bar, 200 μm. n=6 per group. Mann-Whitney *U* tests were used. *P<0.05 vs Scramble. **F**, Quantification of fluorescent staining of reactive oxygen species (ROS) in HAECs transfected with si-GPR35, using scramble oligo as control. Representative images are shown. Scale bar, 200 μm. n=6 per group. Mann-Whitney *U* tests were used. *P<0.05 vs Scramble. In all the figures, horizontal lines show mean±SD.

As shown in Figure 3A, GPR35^KO^ MAECs possessed higher phosphorylated eNOS (p-eNOS^Ser1177^) levels compared with GPR35^WT^ MAECs (n=7 in GPR35^WT^, n=8 in GPR35^KO^, Mann-Whitney *U* test *P*=0.009). In HAECs, the knockdown of GPR35 by Ad-sh-GPR35 significantly increased eNOS phosphorylation (n=6, Mann-Whitney *U* test, *P*=0.002 for p-eNOS^Ser1174^, and *P*=0.002 for the ratio of p-eNOS^Ser1177^/eNOS; Figure 3B). Conversely, GPR35 overexpression by Ad-GPR35 attenuated eNOS phosphorylation (Figure 3C, n=6, Mann-Whitney *U* test *P*=0.026 for the ratio of p-eNOS^Ser1177^/eNOS). Furthermore, migration assay indicated that the enhanced migration induced by GPR35 siRNA was abolished by simultaneous eNOS siRNA (Figure 3D, the left, n=5, Mann-Whitney *U* test Hommel’s adjusted *P*=0.032 for Scramble versus GPR35 siRNA; Mann-Whitney *U* test Hommel’s adjusted *P*=0.032 for Scramble versus eNOS siRNA in GPR35 siRNA groups). The proliferation of HAECs was not impacted (Figure 3D, the right).

Meanwhile, key molecules that modulate eNOS activation in ECs,^32^ such as the PI3K (phosphoinositide 3-kinase)/AKT pathway, PKA (protein kinase A)/adenylate cyclase pathway, and CaMKII (calmodulin kinase II),^8^ and the clamping from Caveolin-1,^33^ were comparable between GPR35^KO^ MAECs and GPR35^WT^ MAECs, or between HAECs with GPR35 knockdown and their controls (Figure S2). However, the redox status in HAECs was altered by GPR35 siRNA because the result from fluorescent staining of intracellular NO and O_2_^−^ clearly demonstrated an increased NO production (Figure 3E, n=5, Mann-Whitney *U* test *P*=0.009) and decreased O_2_^−^ formation (Figure 3F, n=6, Mann-Whitney *U* test *P*=0.026), suggesting that the increased NO availability in ECs with GPR35 knockdown was related to the suppression of oxidative stress.

### GPR35 Depletion Activates GCH1-Mediated BH4 Biosynthesis

**Figure 4.**
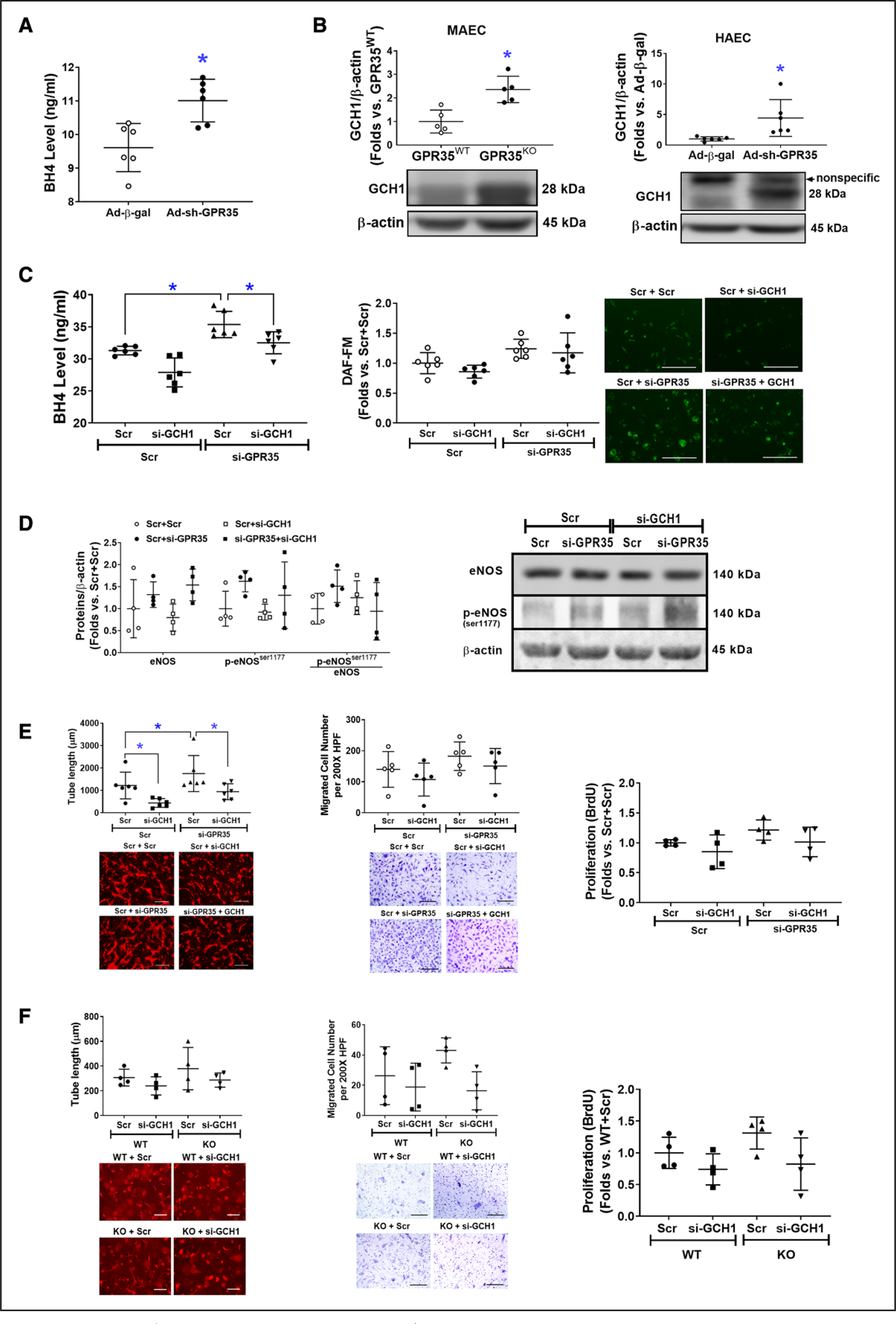
Increased eNOS (endothelial nitric oxide synthase) activation is associated with high expression levels of GCH1 (guanosine triphosphate cyclohydrolase I) protein. **A**, Tetrahydrobiopterin (BH4) levels in the culture medium of HAECs infected with Ad-sh-GPR35 (G-protein–coupled receptor) and Ad-*β*-gal. n=6 per group. Mann-Whitney *U* tests were used. *P<0.05. **B**, Protein expression of GCH1 in GPR35^WT^ and GPR35^KO^ mouse aortic endothelial cells (MAECs; **left**), and in HAECs transfected with Ad-sh-GPR35 or Ad-*β*-gal (**right**). n=5 per group in MAECs, n=6 in HAECs. Mann-Whitney *U* tests were used. *P<0.05 between the 2 groups. Representative bands of Western blot are shown below the quantification analysis. **C**, In HAECs transfected with si-GPR35 and si-GCH1, or scramble oligo as control, BH4 levels in the culture medium of HAECs are shown in the **left** and the intracellular NO levels are shown in the **middle** and the **right**. n=6 per group. Kruskal-Wallis tests were done followed by Mann-Whitney *U* tests with Hommel’s adjustment. *P<0.05 between the 2 groups in post hoc comparisons. Representative images of NO staining are shown next to the dot-plot figure. Scale bar=200 μm. **D**, The eNOS, p-eNOS^ser1177^, and p-eNOS^ser1177^/eNOS in HAECs transfected with si-GPR35 and si-GCH1, or scramble oligo as control. Representative bands of Western blot are shown next to the quantifications. Kruskal-Wallis tests were done but did not reach statistical significance. **E**, Functional assays including 3-dimensional (3D) tube formation (**left**), migration (**middle**), and proliferation by BrdU assay (**right**) in HAECs transfected with si-GPR35 si-GCH1, using scramble oligo as control. Representative images are shown beneath the quantification. Scale bar=200 μm. n=6 per group in tube formation, n=5 per group in migration, n=4 per group in proliferation. Kruskal-Wallis tests were done, followed by Mann-Whitney *U* tests with Hommel’s adjustment. *P<0.05 between the 2 groups in post hoc comparisons. Scale bar=200 μm. **F**, Functional assays including 3D tube formation (**left**), migration (**middle**), and proliferation by BrdU assay (**right**) in GRP35^WT^ and GPR35^KO^ MAECs transfected with si-GCH1 or scramble oligo as control. Representative pictures are shown beneath the qualifications. Scale bar=200 μm. n=6 per group in tube formation, n=4 per group in migration and proliferation. Kruskal-Wallis tests were done but did not reach statistical significance. In all the figures, horizontal lines show mean±SD.

The BH4 levels were significantly increased by GPR35 shRNA (Figure 4A, n=6, Mann-Whitney *U* test *P*=0.009). We observed an elevated GCH1 protein level in GPR35^KO^ MAECs and HAECs with GPR35 shRNA (Figure 4B, right, n=6, Mann-Whitney *U* test *P*=0.008; right, n=6, Mann-Whitney *U* test *P*=0.002), without mRNA changes (Figure S3), suggesting that GPR35 regulates GCH1 at posttranscriptional levels. The increased BH4 levels by GPR35 knockdown were blunted by simultaneously knocking down GCH1 (Figure 4C, left, n=6, Mann-Whitney *U* test Hommel’s adjusted *P*=0.002 for Scramble versus GPR35 siRNA; Mann-Whitney *U* test Hommel’s adjusted *P*=0.026 for Scramble versus GCH1 siRNA in GPR35 siRNA groups), although the NO levels were not significantly impacted upon similar treatments (Figure 4C, the middle and right). Meanwhile, the eNOS protein and phosphorylation were measured by Western blot. As shown in Figure 4D, the p-eNOS^Ser1177^ was not altered by knocking down GCH1 in HAECs with low GPR35. Based on these observations, we think that the regulation of eNOS phosphorylation by GPR35 is independent of the GCH1-mediated BH4 biosynthesis. However, GCH1 participated in GPR35 suppression-induced increase in EC angiogenesis because knocking down GCH1 significantly abolished the 3D tube formation in HAECs with low GPR35 (Figure 4E, left, n=6, Mann-Whitney *U* test Hommel’s adjusted *P*=0.026 for Scramble versus GCH1 siRNA in GPR35 siRNA groups), with no significant impact on migration (Figure 4E, the middle) or proliferation (Figure 4E, the right). Meanwhile, there was a nonsignificant tendency that the knocking down GCH1 compromised the tube formation in GPR35^KO^ ECs (Figure 4F, left, n=6, Mann-Whitney *U* test Hommel’s adjusted *P*=0.082 for Scramble versus GCH1 siRNA in GPR35^KO^ groups). The cell migration (Figure 4F, middle) and proliferation (Figure 4F, right) were not significantly impacted by knocking down GCH1. Taken together, our data suggest that GCH1-mediated BH4 biosynthesis at least partially accounts for GPR35-modulated EC function in vitro.

### Male GPR35^KO^ Mice Have Lower Basal BP Levels With Enhanced Endothelium-Dependent Vasodilation

**Figure 5.**
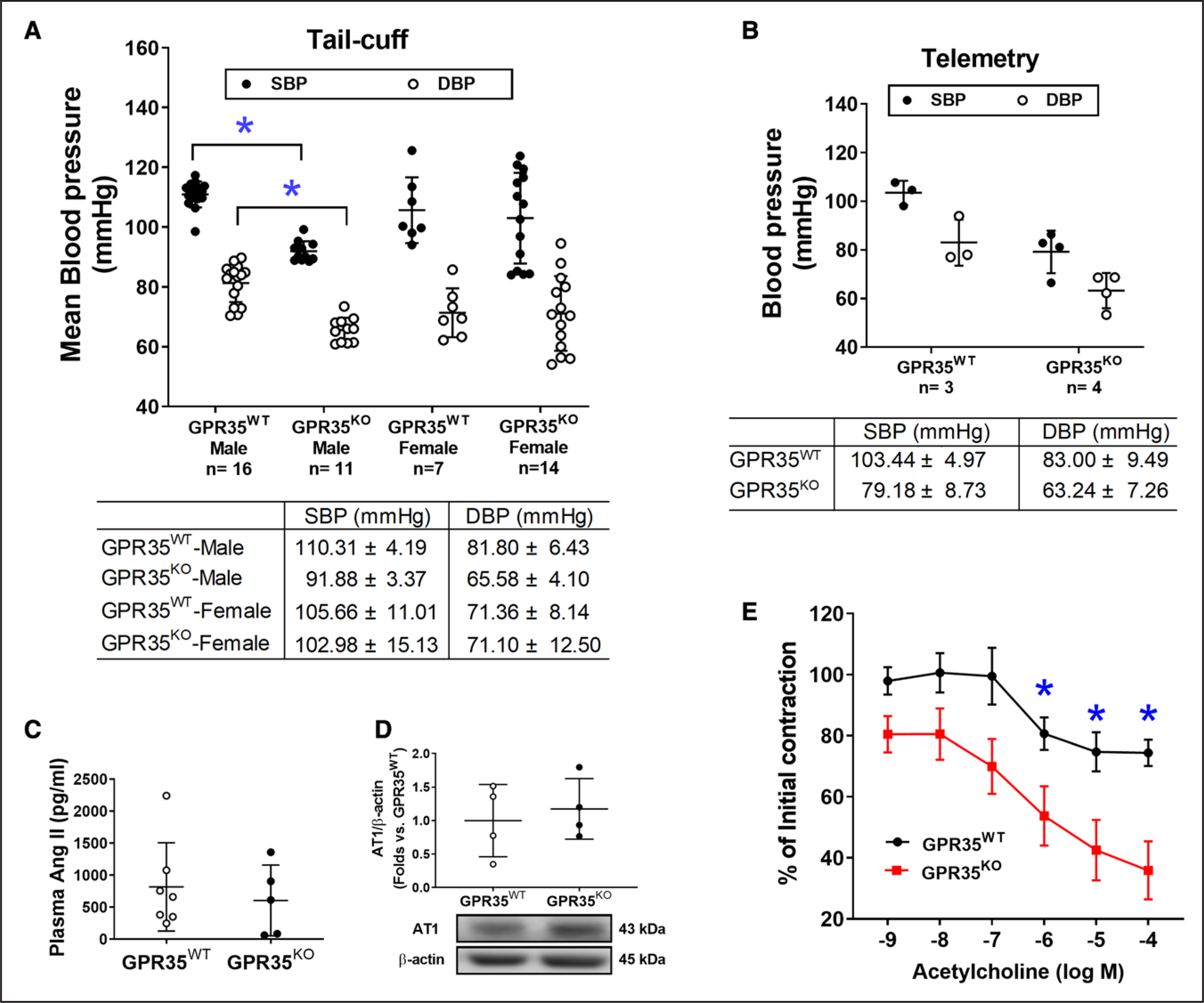
Blood pressure (BP) and endothelium-dependent vasodilation in GPR35^WT^ (G-protein–coupled receptor 35 wild-type control) and GPR35^KO^ mice under physiological conditions. **A**, Systolic blood pressure (SBP) and diastolic blood pressure (DBP) of GPR35^WT^ and GPR35^KO^ adult male and female mice measured by tail-cuff method. The n numbers are shown underneath each group on *x* axis. The mean values of each group are shown underneath the dot plot. One-way ANOVA test was done, then the pairs of focused groups were tested with a 2-sample *t* test and *P* values were adjusted with Hommel’s method. *P<0.05 vs GPR35^WT^ male. **B**, SBP and DBP of GPR35^WT^ and GPR35^KO^ male mice by implanted telemetry transmitter. n=3 in GPR35^WT^, n=4 in GPR35^KO^. Statistical analysis was not performed due to the small sample size. **C**, Plasma Ang II (angiotensin II) levels in GPR35^WT^ and GPR35^KO^ male mice. n=7 in GPR35^WT^, n=5 in GPR35^KO^. Mann-Whitney *U* test did not reach statistical significance. **D**, The AT_1_ (Ang II receptor 1) protein expression in mouse aortic endothelial cells (MAECs) from GPR35^WT^ and GPR35^KO^ male mice. n=4 per group. Mann-Whitney *U* test did not reach statistical significance. Representative bands are shown underneath the dot plot. **E**, Endothelium-dependent vasocontraction of aorta isolated from GPR35^KO^ vs GPR35^WT^ male mice expressed as % initial contraction induced by phenylephrine. n=6. *FDR adjusted Mann-Whitney test *Q* value<0.05. In all the figures, horizontal lines show mean±SD.

The BP profiles of GPR35^WT^ and GPR35^KO^ mice under basal conditions were measured using noninvasive tail-cuff and implanted telemetry methods. Our results suggested that adult male GPR35^KO^ mice showed significantly reduced systolic BP (SBP) by 18 mm Hg (95% CI, −22 to −15 mm Hg) and diastolic BP (DBP) by 16 mm Hg (95% CI, −20 to −11 mm Hg), compared with GPR35^WT^ mice (Figure 5A, measured by the tail-cuff method, 2-sample *t* test Hommel’s adjusted *P*<0.0001). A limited number of animals had BPs assessed by telemetry to verify the findings by the tail-cuff method and showed that the BP levels in GPR35^KO^ mice were 79/63 mm Hg and that the BP levels in GPR35^WT^ mice were 103/85 mm Hg. The difference between the 2 group means was observed (Figure 5B), statistical significance was not evaluated due to the small sample size. Notably, GPR35 deletion did not alter BP in the females but only in the males (Figure 5A). Meanwhile, GPR35^KO^ females showed Bradycardia compared with GPR35^KO^ males, probably due to the baroregulation (Figure S4). Because the renin-angiotensin-aldosterone system plays a significant role in the BP regulation,^34^ we detected plasma Ang II levels in male GPR35^KO^ and GPR35^WT^ mice using ELISA method (MyBioSources, San Diego, CA). Our results indicated that Ang II levels were comparable between GPR35^KO^ and GPR35^WT^ mice (Figure 5C). The AT_1_ (Ang II receptor 1), which mediates most of the biological effects of Ang II,^34^ was also comparable between GPR35^KO^ and GPR35^WT^ mice (Figure 5D). These data suggest that the modulation of BP by GPR35 may not depend on the renin-angiotensin-aldosterone system system. The potentially beneficial effects of GPR35 deletion on endothelial function were studied in the acetylcholine-induced endothelium-dependent vasodilation using aortas from GPR35^KO^ and GPR35^WT^ mice. Maximum dilations to acetylcholine in GPR35^KO^ mice and GPR35^WT^ mice were ≈75% and ≈45% of the preconstriction levels, respectively (Figure 5E). Compared with GPR35^WT^ vessels, the GPR35^KO^ vessels started showing statistically significant dilation in response to acetylcholine at the concentration of 10^−6^ mol/L (false discovery rate adjusted Mann-Whitney test *Q* value=0.036), suggesting that the deletion of GPR35 improved endothelium-dependent vasodilation, which may contribute to the low BP profiles in GPR35^KO^ mice.

### GPR35 Deletion Prevents the Elevation of BP in DOCA-Salt Hypertension Model in Male Mice

**Figure 6.**
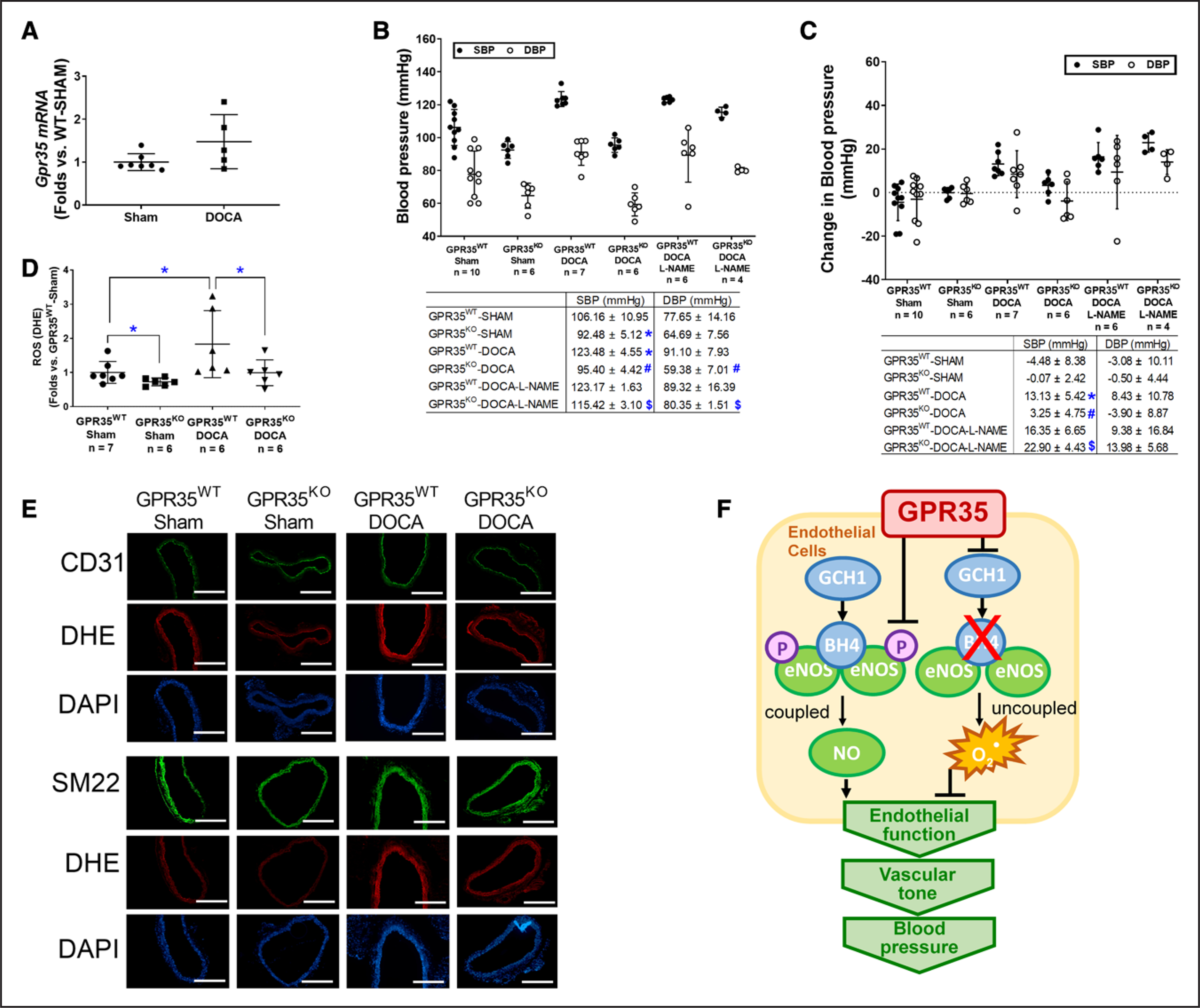
Mice with GPR35 (G-protein–coupled receptor 35) deletion showed resistance to blood pressure elevation and protection of endothelial function after deoxycorticosterone acetate (DOCA)-salt treatment. **A**, The *Gpr35* mRNA level in mouse aortic endothelial cells (MAECs) isolated from GPR35^WT^ mice received DOCA-salt or sham treatment. n=5 in sham group, n=7 in DOCA group. Mann-Whitney *U* test did not reach statistical significance. **B**, The systolic blood pressure (SBP) and diastolic blood pressure (DBP) at the end of the experiment (BP_week 4_) in the animals receiving sham procedure, DOCA-salt procedure, or DOCA-salt with N^G^-nitro-L-arginine methyl ester hydrochloride (L-NAME). The n numbers are shown underneath each group on *x* axis. The mean values of each group are shown underneath the dot plot. One-way ANOVA test was done, then the pairs of focused groups were tested with a 2-sample *t* test and *P* values were adjusted with Hommel method. The symbols denote significant difference found between the 2 groups in post hoc comparisons. *P<0.05 vs GPR35^WT^-Sham, #*P*<0.05 vs GP35^KO^-Sham, $*P*<0.05 vs GPR35^KO^-DOCA. **C**, Change in SBP and DBP in male GPR35^KO^ and male GPR35^WT^ mice after sham, DOCA-salt, or DOCA-salt with L-NAME procedures (BP_week 4_−BP_week 0_). The n numbers are shown underneath each group on *x* axis. The mean values of each group are shown underneath the dot plot. One-way ANOVA test was done, and if significant, pairs of focused groups were tested with a 2-sample *t* test and *P* values were adjusted with Hommel method. The symbols denote significant difference found between the 2 groups in post hoc comparisons. *P<0.05 vs GPR35^WT^-Sham between the 2 groups, #*P*<0.05 vs GP35^KO^-Sham, $*P*<0.05 vs GPR35^KO^-DOCA. **D**, Quantification of reactive oxygen species by dihydroethidium staining in aortas isolated from GPR35^WT^ and GPR35^KO^ mice with DOCA-salt or Sham treatment. The n numbers are shown underneath each group on *x* axis. Kruskal-Wallis tests were done, followed by Mann-Whitney *U* tests with Hommel adjustment. *P<0.05 between the 2 groups in post hoc comparisons. **E**, Representative images of aortas isolated from GPR35^WT^ or GPR35^KO^ mice received DOCA-salt or Sham treatment. Aortas were stained with dihydroethidium, DAPI, and EC marker CD31 or smooth muscle cell marker SM22. Scale bar=400 μm. In all the figures, horizontal lines show mean±SD. **F**, The proposed mechanism by which GPR35 participates in the regulation of endothelial function and BP.

The male GPR35^KO^ and GPR35^WT^ mice were rendered hypertensive by DOCA-salt procedure, using the sham procedure as controls. The GPR35 mRNA expression in MAECs from male GPR35^WT^-DOCA mice was comparable to MAECs from male GPR35^WT^-sham mice (Figure 6A). As shown in Figure 6B and 6C, the male GPR35^WT^ mice with DOCA-salt procedure had higher SBP levels than the male sham-operated GPR35^WT^ mice (measured by tail-cuff method, 123 versus 106 mm Hg, 2-sample *t* test Hommel’s adjusted *P*=0.0056), by an average increase of ≈17 mm Hg (95% CI, 9.90–25.32 mm Hg, 2-sample *t* test Hommel’s adjusted *P*=0.001), while the changes in DBP levels in the 2 groups were not statistically significant. On the contrary, in male GPR35^KO^ mice receiving DOCA-salt procedure, both SBP and DBP levels (95/59 mm Hg) remained comparable to that in male sham-operated GPR35^KO^ mice. Both SBP and DBP levels in male GPR35^KO^ mice receiving DOCA-salt procedure were significantly lower than that in male GPR35^WT^ mice receiving DOCA-salt procedure (95 versus 123 mm Hg in SBP, 2-sample *t* test Hommel’s adjusted *P*=0.020; 59 versus 91 mm Hg in DBP, 2-sample *t* test Hommel’s adjusted *P*=0.0006) by ≈23 mm Hg in SBP (95% CI, −33.56 to −22.55 mm Hg) and ≈32 mm Hg in DBP (95% CI, −40.94 to −22.49 mm Hg). The NO synthase inhibitor L-NAME did not further increase the BP levels in GPR35^WT^ mice receiving DOCA-salt procedure. However, L-NAME significantly increased BP levels in GPR35^KO^ mice receiving DOCA-salt procedure (GPR35^KO^-DOCA + L-NAME versus GPR35^KO^-DOCA) by 19 mm Hg in SBP (95% CI, 12.75–26.55 mm Hg, 2-sample *t* test Hommel’s adjusted *P*=0.001) and 16 mm Hg in DBP (95% CI, 6.22–29.53 mm Hg, 2-sample *t* test Hommel’s adjusted *P*=0.0456), suggesting that the inhibition of eNOS abolished GPR35 deletion-induced the protection of BP levels. This evidence strongly suggests that enhanced eNOS activity plays a vital role in GPR35 regulated vascular tone and BP levels. Meanwhile, the O_2_^−^ level in aortas was evaluated by dihydroethidium staining at the end of the experiment. As shown in Figure 6D and 6E, the aortas from DOCA-salt-operated GPR35^WT^ mice possess high levels of O_2_^−^ compared with those from the sham-operated GPR35^WT^ mice. In aortas from DOCA-salt-operated GPR35^KO^ mice, the O_2_^−^ level was similar to that in the sham-operated GPR35^KO^ mice and much lower than that in DOCA-salt-operated GPR35^WT^ mice (n=7 in GPR35^WT^ sham, n=6 in all other 3 groups, Mann-Whitney *U* test Hommel’s adjusted *P*=0.0411 between GPR35^KO^-DOCA and GPR35^WT^-DOCA), suggesting that the genetic deletion of GPR35 suppressed oxidative stress in vessel wall induced by the DOCA-salt procedure. Meanwhile, we costained the aortic segments with reactive oxygen species indicator dihydroethidium and either EC marker CD31 or smooth muscle cell marker SM22 (Figure 6E). The result showed that both EC and smooth muscle cell layers displayed lowered reactive oxygen species levels in aortas from GPR35^KO^ mice compared with aortas from GPR35^WT^ mice, under the normal physical condition and in the hypertension model, indicating that deletion of GPR35 in smooth muscle cells might also help to reduce the oxidative stress in the vascular wall, which can potentially improve vasodilation.

## DISCUSSION

EC function is critical to vascular homeostasis and neovascularization. In this study, we reported that the deletion of GPR35 induced augmentation of EC functions in vitro, enhanced endothelium-mediated vasodilation in isolated vessels, and prevented BP elevation in the DOCA-salt model in vivo. Furthermore, our data revealed an inhibitory role of GPR35 on eNOS activation and GCH1-mediated BH4 synthesis (Figure 6F). We think that this study provides fresh insights highlighting the therapeutic potential of targeting GPR35 to prevent and treat hypertension.

The maintenance of vascular homeostasis requires a series of orchestrated EC activities. Our study has demonstrated that gene deletion or knockdown of GPR35 in aortic ECs improved migration, tube formation, and proliferation in vitro (Figure 1), suggesting an inhibitory role of GPR35 in EC activities. This study’s in vitro findings align with the robust vasodilation responses in isolated GPR35^KO^ aortas (Figure 5E). Previous reports have demonstrated that endothelial BH4 availability is a pivotal regulator of eNOS activity and enzymatic coupling, but there have been mixed conclusions on the eNOS phosphorylation modulated by BH4. Some studies suggested that BH4 enhances eNOS phosphorylation,^35,36^ while others indicate BH4 engages eNOS in producing NO without altering eNOS phosphorylation.^37–39^ Our data agreed with the latter notion because the eNOS phosphorylation was not changed by GCH1 siRNA (Figure 4D). These observations suggest that the GPR35 inhibition-induced enhancements of BH4 biosynthesis and eNOS phosphorylation are 2 independent mechanisms to ensure NO production in ECs. The molecular basis for the interactions between GPR35 and GCH1 or eNOS is unknown. Several reports suggest that GPR35 binds to Gα_i/o_, Gα_q/11_ with various downstream pathways,^40,41^ including Cyclic adenosine monophosphate/PKA/Akt/mitogen-activated protein kinase (via Gα_i/o)_ and Phospholipase C ß/diacylglycerol/Ca^2+^ (via Gα_q/11_).^42^ In our system, PKA/PI3K/Akt and CaMKII were not altered by GPR35 inhibition (Figure S2A and S2B). We did not find the regulation of GPR35 on the clamping effect of caveolin-1 to eNOS, either (Figure S2C and S2D). However, it is reported that GPR35 couples with G_α12/13_ and activates RhoA/Rho-kinase pathways in Human embryonic kidney 293 cells.^43^ Rhokinase deletion enhances endothelium-dependent vasodilation in an experimental hyperglycemic animal model.^44^ Furthermore, RhoA mediates Ang II–induced impairment of BH4 synthesis in ECs.^39^ Whether GPR35 binds with RhoA to regulate EC functions requires further verification. The current study has established the functional parameters to assess the actions of GPR35 on endothelial function in the primarily cultured ECs and in animal studies, which provides the advantage of identifying the intracellular signaling pathways of GPR35 in pathophysiologically relevant settings. The exploration of GPR35 binding partners and their secondary messengers in ECs will be our subsequent investigation.

ECs with GPR35 inhibition demonstrated low oxidative stress status (Figure 3F). While our data have suggested the eNOS uncoupling due to deficient BH4 as a significant contributor (Figure 3E), other mechanisms might be involved. Previous reports suggest that nicotinamide adenine dinucleotide phosphate oxidase is one of the major sources of O^2−^ in the vasculature.^45^ We detected nicotinamide adenine dinucleotide phosphate subunits, such as Nox2, Nox4, p47^phox^, and p22^phox^ protein expressions in GPR35^KO^ ECs versus GPR^WT^ ECs. The results indicated that p47^phox^ was downregulated in GPR35^KO^ ECs (Western blot, Figure S5), suggesting the nicotinamide adenine dinucleotide phosphate oxidase may participate in the GPR35 pathway. In future studies, we expect to use high throughput proteomic profiling to identify molecules controlled by GPR35. However, given the evidence showing the inhibitory effects of GPR35 on EC activities and the regulation of GPR35 on eNOS and GCH1, it is highly likely that eNOS uncoupling due to deficient GCH1-mediated BH4 biosynthesis is an essential downstream mechanism for GPR35-regulated angiogenesis.

In this study, we observed that male GPR35^KO^ mice displayed lower BP levels than male GPR35^WT^ mice using tail-cuff (Figure 5A) and telemetry methods (Figure 5B). Controversial findings on the role of GPR35 in the regulation of BP levels were reported. Min et al^20^ observed a 37 mm Hg increase in left ventricular SBP in GPR35^KO^ mice compared with GPR35^KO^ mice (sex not specified in the report). This might be due to the differences in the experimental techniques. Min et al^20^ used direct Millar catheterization of the left ventricle for hemodynamic recording in animals under terminal anesthesia. In our study, the arterial BP was measured in conscious, free-moving animals in their home cages. Anesthesia can be a significant contributor to BP variation because GPR35 is highly expressed in sympathetic neurons and regulates excitability and synaptic release critical to hemodynamic changes under anesthesia.^46^ In another report, Divorty et al^22^ reported that male GPR35^KO^ mice were resistant to Ang II–induced hypertension. However, in their study, the BP profiles were comparable between male GPR35^KO^ and male GPR35^WT^ mice under basal conditions, and the plasma Ang II levels were not provided. We found a significantly lower BP profile in male GPR35^KO^ mice than in male GPR35^WT^ mice without the difference in plasma Ang II levels (Figure 5C) or endothelial AT_1_ receptor expression (Figure 5D). Experimental hypertension induced by exogenous Ang II should not be equated with hypertension due to endogenous elevation in plasma Ang II.^47–49^ In a pilot study, we stimulated the MAECs with Ang II in vitro and evaluated EC activities, including 2D network formation, migration, and proliferation (Figure S6). The results demonstrated that the extent of Ang II–induced reductions of 2D network was comparable between GPR35^KO^ and GPR35^WT^ MAECs (Figure S6A). The changes in migration and proliferation were not statistically significant (Figure S6B and S6C). Based on these observations, we have concluded that Ang II is not the primary mechanism mediating the effects of GPR35 on EC activities in vitro. However, since we did not test the vasoconstriction response of GPR35^KO^ vessels to Ang II or the renin-angiotensin-aldosterone system component levels in tissues regulating BP and body fluid homeostasis, we cannot rule out the participation of the renin-angiotensin-aldosterone system system in GPR35-regulated hemodynamic changes. Future studies will be needed to dissect the roles of GPR35 in the cardiac, renal, and sympathetic control over BP.

We discovered dramatically different BP profiles between male and female GPR35^KO^ mice under basal conditions using the tail-cuff method (Figure 5A). Female GRP35^KO^ mice had comparable heart rates and BP levels to female GPR35^WT^ mice (Figure S4), while female GPR35^KO^ mice had lower heart rates than male GPR35^WT^ mice (1-way ANOVA test followed by 2-sample *t* test Hommel’s adjusted *P*=0.0018). The low heart rates in female GPR35^KO^ mice might be due to altered baroreflex mechanisms, an interesting phenomenon to be investigated. In addition, ECs isolated from female GPR35^KO^ and GPR35^WT^ mice did not show any difference in cell migration, proliferation, or eNOS phosphorylation (Figure S7), suggesting that there is sexual dimorphism in GPR35 regulated EC function. Notably, the growth factors and hormonal factors in the in vitro culture environment are unified, while those in the in vivo environment differ considerably between female and male mice. This may contribute to the discrepancies in the observations between in vitro and in vivo experiments. However, the underlying mechanism for the sex difference in GPR35 regulated hemodynamics remains unclear. Estrogen has confounding protective effects on vasodilatory and metabolic pathways in cardiovascular components.^50^ The hormonal variation in estrous cycles can potentially interfere with the regulation of GPR35 on BP. Therefore, it would be necessary to investigate such sex-specific BP regulation by GPR35 in future studies in which the estrous cycles in female animals are monitored.

We recognized the limitations of our studies. First, hypertension is a complex syndrome involving multiple organs and systems. Metabolic and local neuronal factors also have substantial effects on local vascular tone. It would be arbitrary to assume that GPR35 regulates hemodynamic changes through a single organ or tissue. Interestingly, it has been reported that GPR35 promotes Na/K-ATPase activity in macrophages and intestinal epithelial cells.^51^ Whether GPR35 also participates in water retention by modulating sodium-potassium pumps in renal tubular epithelial cells remains to be tested. Interestingly, a recent report has shown that the loop diuretic drugs bumetanide and furosemide can activate human GPR35 but not rodent GPR35.^52^ Research efforts into GPR35 in multiple systems across species may help identify better the novel therapeutic potential of GPR35 in preventing hypertension. Nonetheless, our studies demonstrated that simultaneous L-NAME treatment in the DOCA-salt hypertension model abolished the protection of GPR35 deletion on BP (Figure 6B and 6C). Based on the evidence, we think that ECs are at least the major cell types targeted by GPR35 to regulate BP. Second, although the evidence of GPR35 expressions in human ECs from hypertensive donors would significantly strengthen the study, we could not do so because it was challenging to find human arterial ECs in a population with hypertension as the only disease state. Most donors have hypertension as a co-morbidity, but these donors have been excluded from our study. Meanwhile, the genome-wide association studies did not show the connection between GPR35 polymorphisms with hypertension. However, this is conceivable because hypertension is a multifactorial disease that the polymorphisms of a single gene might not yield a significant contribution to the disease state. An alternative way is to relate GPR35 phenotypes to the markers for endothelial (dys) function, such as flow-mediated endothelium-dependent vasodilation or the circulating endothelial microparticles in the human population.^53,54^ This will provide the prognostic value of GPR35 in the setting of cardiovascular and metabolic disorders. Third, our study did not test the functional consequence of GPR35 agonism/antagonism in the in vitro and in vivo studies. The genetic techniques were used because many compounds targeting GPR35 have shown species-selectivity or partial agonism/antagonism, masking the actions of GPR35 in modulating EC function. By providing the proof-of-concept evidence and the underlying molecular mechanisms for targeting GPR35 in the endothelium in this study, we hope to advocate for more efforts in the development of pharmacological or genetic agents for the GPR35 study. Probing the pharmacological and pathophysiological roles of GPR35 in the cardiovascular system will be a fruitful future research area.

## PERSPECTIVES

We think that data accrued in our studies open a new avenue to the preservation of endothelial function by targeting GPR35 as a potentially promising approach. A sex dimorphism has been observed in GPR35’s actions on endothelial function and BP regulation. This warrants future investigations. Given the involvement of GPR35 in a wide range of physiological and pathophysiological processes, future investigations into the pharmacological characteristics of GPR35 agonism and antagonism will provide a unique opportunity to probe for the functional importance of GPR35 in disease-relevant context using either human-derived cells or animal models.

## Supplementary Material

Supplementary Material

## Novelty and Significance

### What Is New?

- Our work is based on an orphan GPR (G-protein–coupled receptor) whose physiological ligand and exact intracellular signaling pathways remain poorly characterized. Currently, it is not known whether elevation of GPR35 plays causal roles in disrupted endothelial function. However, our data indicate that genetic deletion of GPR35 augments endothelial cell functions in vitro and vasodilation in male animals in vivo. This contributes to maintaining normal blood pressure in an experimental hypertensive animal model.
- In addition, although previous studies have established that tetrahydrobiopterin is an essential cofactor for eNOS (endothelial nitric oxide synthase), the upstream regulatory mechanisms for GCH1 (guanosine triphosphate cyclohydrolase I) are missing. We have demonstrated that GPR35 inhibits GCH1 expression at protein levels, providing novel insights into how GCH1 is regulated.
- The modulation of GPR35 on blood pressure is observed in male animals but is absent in female animals. These male-biased finding in GPR35’s action on hemodynamic regulation is intriguing, but future studies are needed to investigate underlying mechanisms.

### What Is Relevant?

- This work is relevant to the human population because our data shows that inhibiting GPR35 protects against blood pressure elevation under a hypertensive mouse model that mimics low-renin hypertension prevalent biochemical phenotype in up to 30% of patients with hypertension, depending on age and race.^55^

### Summary

This study uncovered novel molecular mechanisms underlying the benefit of GPR35 inhibition on endothelial function and provide proof-of-concept evidence for GPR35-targeted therapeutic strategies in regulating blood pressure, paving the avenue for future drug discoveries based on this GPR in cardiovascular and metabolic disorders.

## Nonstandard Abbreviations and Acronyms

3D: 3-dimensional
Ang II: angiotensin II
**AT**_1_: Ang II receptor 1
BH4: tetrahydrobiopterin
BP: blood pressure
CaMKII: calmodulin kinase II
DBP: diastolic BP
DOCA: deoxycorticosterone acetate
EC: endothelial cell
eNOS: endothelial nitric oxide synthase
GCH1: guanosine triphosphate cyclohydrolase I
GFP: green fluorescent protein
GPR35: G-protein–coupled receptor 35
GPR35^KO^: GPR35 knockout
GPR35^WT^: GPR35 wild-type control
HAECs: human aortic ECs
L-NAME: N^G^-nitro-L-arginine methyl ester hydrochloride
MAECs: mouse aortic ECs
NO: nitric oxide
PI3K: phosphoinositide 3-kinase
PKA: Protein kinase A
SBP: systolic BP
