# Supplementary material for: Novel Role of GPR35 (G-Protein–Coupled Receptor 35) in the Regulation of Endothelial Cell Function and Blood Pressure
Source: Hypertension. 2021 Jul 19;78(3):816–30. doi: 10.1161/HYPERTENSIONAHA.120.15423 (PMC8357038; doi:10.1161/HYPERTENSIONAHA.120.15423)
Supplement: Supplementary file 1 [file hyp-78-816-s002.docx]

**Supplemental Materials**

**Title:  A novel role of G protein-coupled receptor 35 (GPR35) in the regulation of endothelial cell function.** 

^1^Hainan Li, MS; ^1^Huong Nguyen, Ph.D.; ^1^Sai Pranathi Meda Venkata, ^1^MS; Jia Yi Koh, BS; ^1,2^Anjaneyulu Kowluru, PhD; ^3^Li Li, Ph.D.; ^2,3 ^Noreen F. Rossi, MD; ^4,5^Wei Chen, Ph.D., ^1,6^Jie-Mei Wang, M.D., Ph.D.*

^1^ Department of Pharmaceutical Sciences, Eugene Applebaum College of Pharmacy and Health Sciences, Wayne State University, Detroit, MI;^ 2^John D. Dingell VA Medical Center, Detroit, MI; ^3^Departments of Internal Medicine, School of Medicine; ^4^Karmanos Cancer Institute; ^5^Department of Oncology, School of Medicine; ^6^Centers for Molecular Medicine and Genetics, Wayne State University, Detroit, MI, USA. 

**Running Title:**  GPR35 in endothelial cell function and blood pressure

*****To whom correspondence should be addressed:

**Jie-Mei Wang**, M.D., Ph.D., Department of Pharmaceutical Sciences, Eugene Applebaum College of Pharmacy & Health Sciences, Wayne State University. Address: 259 Mack Ave, 3122 Applebaum Building, Detroit, MI 48201, USA. Tel: +1 313 577 1715; Fax: +1 313 577 2133; E-mail: [jiemei.wang@wayne.edu](mailto:jiemei.wang@wayne.edu). 

**Expanded Materials & Methods**

**Animal procedures**

GPR35 knockout mice (GPR35^KO^, on the C57BL/6 background) were provided by Wellcome Trust Sanger Institute (Cambridge, England)^1, 2^. GPR35^KO^ mice were bred with specific pathogen-free male C57BL/6 mice procured from The Jackson Laboratory (Bar Harbor, ME) to generate GPR35^KO^ and their age- and gender-matched wild type control (GPR35^WT^). The animal experiment was originally planned with 6 mice in each group in each batch.   During the revisions, we expanded the sample size with all available animals in the colony to the existing ones.  The final treatment groups have different sample sizes due to the availability of animals in the colony.  All animal experiments were performed in accordance with the guidelines of the National Institutes of Health *Guide for the Care and Use of Laboratory Animals,* and approved by the Wayne State University Institutional Animal Care and Use Committee. 

**Mouse primary aortic endothelial cell culture**

Primary mouse aortic endothelial cells (MAECs) were cultured from GPR35^KO^ mice and GPR35^WT^ mice. For the initial culturing, a collagen mixture was prepared by the addition of 400 μL of collagen type-1 rat tail (Corning, 354236), 1.388 mL of Dulbecco’s modified Eagles’s medium (DMEM), 2 μL of 5M NaOH. An amount of 760 μL of the collagen mixture was added to a 35 mm dish and allowed to solidify at 37°C for 20 min. The aorta segments were placed lumen-side-down onto the collagen gel in DMEM containing 100 μg/mL Endothelial Cell Growth Supplements (ECGS, Sigma Aldrich), 10% FBS, and 1% Penicillin/Streptomycin. The segments were removed on Day 4. After 3-4 days when outgrowth of cells was observed, the MAECs were passaged using trypsin and reseeded on 0.1% gelatin-coated cell culture plates. MAECs at passages 3-5 were used for the experiments. MAECs were characterized by *Ulex*-lectin (Sigma, L9006) binding and *Dil*-ac-LDL (Thermo Fisher Scientific, L3484) uptake by direct fluorescent staining, immunofluorescent staining of EC cell surface markers CD31, VE-cadherin (CD144), and endothelial nitric oxide synthase (eNOS) as a typical endothelial functional protein.

**Human aortic endothelial cells (HAECs)** ***in vitro*** **culture** 

HAECs were purchase from Lonza (CC-2535) and cultured in endothelial growth media (EGM-2, Lonza CC-3162) at 37 °C, 5% CO_2_. HAECs were used between passages 4 and 6, and all experiments were performed using HAECs from different donors (donor information: n=6, age 53.67 ± 1.12 years, male/female = 5:1). In the original submission, all human cell experiments were initiated with 4 donors. We were able to obtain two additional donors during the revision for some of the specific human cell experiments.  Furthermore, the final sample size varies due to insufficient cell numbers harvested from some donors. Therefore, in some experiments, the sample size was less than 6.

**Gene manipulation treatments** 

For infection of an adenovirus to HAECs, adenovirus carrying human *GPR35* (ENSG00000178623, Ad-GPR35, Vector Biolabs, ADV-210350), shRNA against *gpr35* (Ad-shGPR35, Vector Biolabs, shADV-210350), human β*-gal* (Ad-β-gal, Vector Biolabs, 1080) or *egfp* gene (Ad-GFP) were infected at a multiplicity of infection (MOI) of 50 MOI for 48 hours as described previously ^3^. For small interfering RNA (siRNA)-mediated gene knockdown, siRNA duplexes against GPR35 (Thermo Fisher) were transfected into HAECs with DharmaFECT transfection reagent I (Dharmacon, T2001-03) according to the protocol of the manufacturer. Non-related scramble oligo was used as a negative control. The cells were harvested for the *in vitro* and *in vivo* experiments after 48 hours of siRNA transfection.

**Cell Functional Assays (tube formation, Migration, and Proliferation)**

Angiogenesis was evaluated by either 2D network formation or by a 3D tube formation assay.  In the tube formation, to prepare 1 mL of collagen/media solution, 340 μL of type I rat tail collagen (Corning, 354236), 76 μL of 10 × M199 (Sigma, M0650), 136 μL serum-free DMEM, 100 μL FBS, and 136 μL of DPBS were mixed on ice. The pH was adjusted to 7.2 with NaOH. 1.8 × 10^6^/mL cells were mixed for a final collagen concentration of 1.25 mg/mL. An amount of 30 μL of collagen/cell mixture was loaded onto a sterilized 5-mm woven nylon mesh ring in a 48-well culture plate. The culture media was added after 1 hour of polymerization of the collagen/cell mixture at 37 °C, 5% CO_2_. The culture media consisted of EBM-2 supplemented with 1% FBS, 30 mg/mL VEGF-A^165^ (Peprotech, 100-20). On day 6-8, the collagen-embedded cells were fixed in 4% formaldehyde and stained with 10 µg/mL FITC conjugated-lectin (Ulex europaeus) (Sigma, L9006). The images were taken at FITC fluorescent channel using EVOS FL Imaging System. The tube formation assays measured the total lengths of networks or tubes in the observed areas using a scale bar as a reference. The data were presented in length (µm). Cell proliferation was evaluated using BrdU Cell Proliferation Assay Kit (Cell Signaling Technology) according to the manufacture’s instruction. Briefly, after the treatments, cells were seeded in a 96-well culture plate (1 X 10^4^ cells/well) for 24 hours. The media were replaced with 1 X BrdU solution and cells were culture for 20 hr. Then the cells were fixed and incubated with detection antibody solution, following by HRP-conjugated secondary antibody solution. After the stop solution was added, the absorbance of each well was read at 450 nm (optical density, OD_450_) with Epoch Microplate Spectrophotometer (BioTek). A mean value of the control group was calculated. The value of each sample was then divided by this value to get the fold-change vs. the control group.  Migration was assessed by a modified Boyden chamber assay. The transwell inserts with 8-μm pore size were coated with 0.5% gelatin. 3×10^4^ cells were loaded into the upper chamber with FBS-free media. The inserts were transferred to the lower chamber in a 24-well plate containing medium with 10% FBS. The cells were incubated for 6 h at 37 °C, 5% CO_2_. Cells that had not migrated from the top of the insert membrane were removed by wet cotton swabs. Cells that migrated to the lower side of the membrane were fixed with 4% paraformaldehyde for 10 min and stained with crystal violet.  The images were taken at phase-contrast channel using EVOS FL Imaging System. The migrated cells were counted in a mean value of 5 different fields at 100X magnification for each sample. The data were presented as the number of migrated cells per high power field (HPF). 

**Western Blot analyses**

For intracellular protein measurement, BMPCs were lysed using Cell Lytic MT lysis buffer (Sigma, C3228) with Protease Inhibitor Cocktail (1:100 v/v, ThermoFisher) for 20 minutes on ice. The cell suspension was sonicated at 20% amplitude for 30 seconds on ice. The protein concentration was determined by Bradford Assay using Quick StartTM Bradford 1 X Dye Reagent (Bio-Rad, 500-0205). Equal amounts of denatured protein (30 μg) were separated by SDS-PAGE on 10% Tris-glycine polyacrylamide gels and transferred to a 0.45-mm polyvinylidene fluoride (PVDF) membrane (Sigma) together with WesternSure Pre-stained Chemiluminescent Protein Ladder (Li-Cor, P/N 926-98000)). Immunoblotting was performed by using antibodies directed against each target molecule: eNOS (#32027, 1:1000), phospho-eNOS (p-eNOS, #9571, 1:1000), GFP (#2956, 1:1000), PKA (#5842, 1:1000), phospho-PI3K p85 (Tyr458)/p55(Tyr199) (#4228, 1:1000), CAMKII (#3362, 1:1000), Akt (#2920, 1:2000), phosphorylated Akt (Ser473) (#4060, 1:2000), GCH1 (NBP1-79771, 1:1000) from NOVUS, PI3K (sc-376641, 1:500), AT_1_ (sc-515884, 1:500) from Santa Cruz. The housekeeping protein was β-actin (#12262, 1:10000, from Cell Signaling). Secondary antibodies included HRP goat anti-mouse IgG (P/N 926-80010, 1:10000) and HRP goat anti-rabbit IgG (P/N 926-80011, 1:6000) from Li-Cor were incubated at room temperature for 1 hour. Membrane-bound antibodies were detected by an enhanced chemiluminescence detection reagent (WesternSure ECL Substrate, Li-Cor, 926-95000). Quantitative analysis of protein levels was analyzed with Image Studio Lite Ver 5.2 (Li-Cor). We detected the intensity of the target protein and the housekeeping protein β-actin of each sample. The target protein expression was divided by β-actin to get the relative abundance (considered as the first delta) in each sample. A mean value of the control group was calculated. The value of each sample was then divided by this value to get the fold-change (considered as the second delta).  The data were presented as the fold-change vs. the control group.  

**Co-Immunoprecipitation**

HAEC lysate was harvested after infected with Ad-sh-GPR35 or Ad-β-gal. A total of 300 µg of protein was used for each pull-down reaction with µg of rabbit anti-eNOS antibody (Cell Signaling, #32027), according to the Pierce® Crosslink Immunoprecipitation Kit manufacturer’s protocol (Thermo Scientific, 26149). Co-immunoprecipitation (co-IP) was done using the Pierce® Crosslink Immunoprecipitation Kit (Thermo Scientific, 26149) according to the manufacturer’s instructions. Briefly, 1 µg of eNOS antibody (Cell Signaling, #32027) was first immobilized for 2 h using AminoLink Plus Coupling Resin. After washing the resin, 300 µg of HAEC lysate was incubated with the resin overnight at 4 °C. The resin was then rewashed, and the protein was eluted by elution buffer. The samples were analyzed by Western blot using Rabbit anti-Caveolin-1 antibody (Cell Signaling, #3267) and rabbit anti-eNOS antibody (Cell Signaling, #32027, 1:1000). A mean value of the control group was calculated. The value of each sample was then divided by this value to get the fold-change (considered as the second delta).  The data were presented as the fold-change vs. the control group.  

**Nitric oxide measurement**

Cytosolic nitric oxide in HAEC was semi-quantified using the fluorescent nitric oxide probe 4-amino-5-methylamino-2′,7′-difluorofluorescein diacetate (DAF-FM DA) (ThermoFisher Scientific, D23842) according to the manufacturer’s protocol. Briefly, after seeding HAECs on NuncTM Lab-TekTM II Chamber SlideTM system (ThermoFisher Scientific, 154453) and transfected with siRNA against GPR35 or scramble control, HAECs were exposed to 1 µM of DAF-FM DA for 15 min at 37°C. Subsequently, HAECs were gently washed twice with PBS to remove the excess dye and further incubate in the dark for 30 minutes at 37°C to convert DAF-FM DA to its active form. Following incubation, HAECs were fixed with 4% PFA for 15 minutes. The slides were then mounted with DAPI Fluoromount-G (SouthernBiotech, 0100-20). The fluorescence of NO was recorded by EVOS FL Imaging System (Thermo Fisher Scientific) at the wavelength 480nm/510nm.  The signal density was analyzed by Image Pro-Plus software (Media Cybernetics, Inc.). A mean value of the control group was calculated. The value of each sample was then divided by this value to get the fold-change vs. the control group. The data were presented as the fold-change vs. the control group.

**Dihydroethidium** **(DHE) fluorescence staining**

The HAECs transfected with siRNA against GPR35 and scramble oligo control were stained with 10 µM of dihydroethidium (DHE, Thermofisher, D11347) for 15 minutes at 37^o^C, and gently washed twice with HBSS. Then the HAECs were counterstained with 10ug/mL Hoechst 33342 solution (BD Pharmingen, 561908) for 5 min and washed with HBSS. The cells were visualized under fluorescent microscope for imaging.   Aortas isolated from GPR35 mice were embedded in OCT. 6 µm sections were cut using a cryostat and transferred to poly-L-lysine coated slides. The aorta sections were stained with 10 µM of DHE for 13 minutes at 37^o^C then gently washed twice with PBS. The sections were then permeabilized by incubating with 1% BSA and 0.4% Trition X-100 in PBS for 20 minutes. 3% BSA in PBS-T was used as blocking buffer to block the non-specific binding for 30 minutes at room temperature. The sections were then incubated with a goat anti-mouse CD31 antibody (R&D, AF3628), in a humidified chamber overnight at 4^o^C, followed by Donkey anti-goat green IgG NorthermLightsTM NL493-conjugated secondary antibody (R&D system, NL-493), or MYH11 (Santa Cruz, sc-6956), followed by an Alexa Fluor 488 goat anti-mouse IgG secondary antibody (Thermo Fisher, A-11001). Sections were then mounted with DAPI Fluoromount-G (SouthernBiotech, 0100-20). In both cell culture and tissue session samples, the fluorescent images were recorded by EVOS FL Imaging System (Thermo Fisher Scientific).  The fluorescent intensity arbitrary units were recorded for each sample. A mean value of the control group was calculated. The value of each sample was then divided by this value to get the fold-change vs. the control group. The data were presented as the fold-change vs. the control group.

**Measurement of tetrahydrobiopterin** 

The levels of tetrahydrobiopterin (BH4) in the media culturing HAECs after treatment were measured by tetrahydrobiopterin ELISA Kit (MyBioSources, MBS283103) according to the manufacturer’s manual. The data were presented in ng/mL). 

**Quantitative real-time PCR**

Total RNA from cells was isolated by miRNeasy Mini Kit (Qiagen, 74106) and then reverse transcribed to cDNA using High Capacity cDNA Reverse Transcription kit (Applied Biosystems, 4368814). For mRNA expression analysis, quantitative real-time PCR (qRT-PCR) was performed using primers synthesized by Integrated DNA Technologies. Amplification and detection of specific gene products were performed with the LightCycler 480 (Roche) System using Fast SYBRTM Green Master Mix (Thermo Fisher Scientific). The Ct value was normalized by subtracting the Ct value of the housekeeping gene *GAPDH*, which gave the ΔCt value for each sample. The mean ΔCt value of the control group was calculated (Mean ΔCt_control). Finally, the relative expression of the target gene for each sample was calculated using the following equation: 2^-(ΔCt_individual - Mean ΔCt_control)^. The data were presented as the fold-change vs. the control group.

**Telemetry transmitter implantation and data acquisition**

Buprenorphine S.R. (1 mg/kg) was administered 1 hour before the surgery. All surgical procedures were conducted using aseptic techniques under isoflurane anesthesia (4% for induction, 1% for maintenance in the mixture of fresh air/O_2_). To measure blood pressure and heart rates, telemetry transmitters (Data Sciences International, Model TA11PA-C10) were implanted. Briefly, a midline incision was made below the neck of the mouse and another incision was made in the dorsal left side behind the scapula. A TA11PA-C10 transmitter was placed subcutaneously along the flank. The pressure-sensing catheter connected with the transmitter was inserted in the left carotid artery near the aortic arch. Mice were housed singly following the telemetry implantation. Hemodynamic measurements were sampled continuously for 1 h on one day for 2 days after a 7-day recovery period. The transmitters were turned on and off by briefly positioning a magnetic device close to the animal. Recordings were started 20 min after turning on the transmitters. The data of blood pressure (mmHg) and heart rates (beats/minutes) from each animal were exported using Ponemah software (Data Sciences International). 

**DOCA-Salt induced hypertensive model construction**

Before the experiment, the systolic, diastolic, and mean arterial blood pressure and heart rate of the mice were measured for 5 consecutive days. The mice were anesthetized with isoflurane in the mixture of fresh oxygen. The left kidney was exteriorized and removed after ligation of the renal artery, vein, and ureter. The mice in the DOCA-Salt group were implanted with a 66-mg DOCA pellet (28-day release, Innovative Research of America) and fed with drinking water containing 1.0% NaCl and 0.2% KCl for 4 weeks. The mice in the sham groups had their left kidneys removed only and were fed with tap water. Mice in L-NAME-Treated groups were administered 0.1g/L L-NAME (Sigma) in drinking water for 4 weeks after the surgery. L-NAME-contained drinking water was replaced twice a week. The blood pressure (mmHg) and heart rate (beats/minute) were monitored on these mice by the non-invasive Tail cuff method (Kent Scientific) once a week for 4 consecutive weeks. 

**Vasorelaxation assay** 

The vasorelaxation assay was performed using *in vitro* wire myography system (Wire Myograph 620M, Danish Myo Technology, Denmark) following the manufacturer’s instructions with a few modifications. Briefly, immediately after euthanasia, the thoracic aortas were dissected, cleaned of excessive connective tissue, and cut into 3 mm ring segments. Aortic rings were then mounted on wire hooks, suspended in the chambers containing 5 mL of warmed physiological saline solution (PSS, NaCl 130 mM, KCl 4.7 mM, KH_2_PO_4_ 1.18 mM, MgSO_4_·7H_2_O 1.17 mM, NaHCO_3_ 24.9 mM, Glucose 5.5 mM, CaCl_2_ 1.6 mM). The chambers were maintained at 37 °C and continuously aerated with 95% O_2_/5% CO_2_. After equilibration for 1 hour at a preload tension of 3 millinewtons (mN), the mechanical and functional properties of the aortic rings were reactivated by incubation with high potassium buffer (NaCl 74.7 mM, KCl 60 mM, KH_2_PO_4_ 1.18 mM, MgSO_4_·7H_2_O 1.17 mM, NaHCO_3_ 24.9 mM, Glucose 5.5 mM, CaCl_2_ 1.6 mM) for 30 minutes, following by washing 3 times with PSS. We pre-constricted the aortic rings with 1 µM of phenylephrine. After a steady-state achieved, the rings were exposed to cumulative concentrations of acetylcholine (ACh, 10^-9^ to 10^-4^ M) with 10 min interval for each concentration to induce relaxant responses. The tension of each vessel segment was recorded by the Myograph 620M system and the response of the vessel tension to each concentration of ACh were calculated and expressed as the percentage of the initial constriction.

**Statistics**

All values are expressed as mean ± SD. For continuous variables that failed Shiparo-Wilk normality tests such as mRNA expression, protein levels, staining quantifications, functional assays, the statistical significance of differences between the two groups was determined with the Mann-Whitney U test. In myograph data, the two groups were tested using muiltiple Mann-Whitney U tests with Benjamini Krieger, and Yekutieli’s adjustment ^4^. When more than two groups of treatments were performed, we used the Kruskal-Wallis test across all the groups, and if significant, we then tested the pairs of our primary interest based on scientific rationale using the Mann-Whitney U test with Hommel’s adjustment for multiple comparisons ^5^. These gatekeeping approaches and the adjustments preserved alpha spending and controlled false positive rate inflation due to multiple hypothesis testing. The significant differences came from post-hoc comparisons of groups were noted. Similarly, for continuous variables that follow the normal distributions, such as BP and heart rates, the statistical significance of differences between the two groups was determined by the student’s *t*-test. When more than two treatment groups were performed, one-way ANOVA was used across all the groups, and if significant, pairs of focused groups were tested with two sample *t*-test and p values were adjusted with Hommel’s method.  A value of p < 0.05 was considered statistically significant. All the statistical analyses were performed using GraphPad Prism 9 (GraphPad Software). The Hommel’s adjustment was performed using R version 3.6.3.

**REFERENCES (All the references have been mentioned in the manuscript text.)**

1.	Skarnes WC, Rosen B, West AP, Koutsourakis M, Bushell W, Iyer V, Mujica AO, Thomas M, Harrow J, Cox T, Jackson D, Severin J, Biggs P, Fu J, Nefedov M, de Jong PJ, Stewart AF, Bradley A. A conditional knockout resource for the genome-wide study of mouse gene function. *Nature*. 2011;474:337-342

2.	White JK, Gerdin AK, Karp NA, Ryder E, Buljan M, Bussell JN, Salisbury J, Clare S, Ingham NJ, Podrini C, Houghton R, Estabel J, Bottomley JR, Melvin DG, Sunter D, Adams NC, Tannahill D, Logan DW, Macarthur DG, Flint J, Mahajan VB, Tsang SH, Smyth I, Watt FM, Skarnes WC, Dougan G, Adams DJ, Ramirez-Solis R, Bradley A, Steel KP. Genome-wide generation and systematic phenotyping of knockout mice reveals new roles for many genes. *Cell*. 2013;154:452-464

3.	Li H, O'Meara M, Zhang X, Zhang K, Seyoum B, Yi Z, Kaufman RJ, Monks TJ, Wang JM. Ameliorating methylglyoxal-induced progenitor cell dysfunction for tissue repair in diabetes. *Diabetes*. 2019;68:1287-1302

4.	Benjamini Y, Krieger AM, Yekutieli D. Adaptive linear step-up procedures that control the false discovery rate. *Biometrika*. 2006;93:491-507

5.	Hommel G. A stagewise rejective multiple test procedure based on a modified bonferroni test. *Biometrika*. 1988;75:383-386

**Figure S1**

**Figure S1. Genetic manipulation of GPR35 in human aortic endothelial cells (HAECs).** The inhibition of GPR35 expression in HAECs was through the infection of adenovirus carrying shRNA against the human *GPR35* gene (Ad-sh-GPR35) with a green fluorescent protein (GFP) tag. The overexpression of GPR35 in HAECs was through the infection of adenovirus carrying the human *GPR35* gene (ENSG00000178632) (Ad-GPR35). (**A**) Overexpression of GFP protein showed successful infection of Ad-sh-GPR35 to HAECs, using adenovirus carrying β-gal (Ad-β-gal) as control. n = 6, ‘*’ denotes p < 0.05. **B,** qPCR confirmed that *GPR35* mRNA expression level in human aortic endothelial cells (HAECs) decreased after infection of Ad-sh-GPR35 and Ad-β-gal. n = 6, ‘*’ denotes p < 0.05. **C**, qPCR confirmed that *GPR35* mRNA expression level in HAECs was increased after infection of Ad-GPR35 and Ad-β-gal. n = 5, ‘*’ denotes p < 0.05. The p values in this figure were determined by the Mann-Whitney U tests. In all the dot-plot figures, horizontal lines show mean ± SD.

**Figure S2**

**Figure S2. The eNOS activity in GPR35 knocked down HAECs may not be regulated by PKA, PI3K/Akt, CaMKII, or Caveolin-1 pathway.** 

(**A**) Western blot analysis of PKA, PI3K, P-PI3K, AKT, P-AKT, CaMKII, and β-actin expression in GPR35^WT^ and GPR35^KO^ MAECs. n = 5 per group. Representative bands are shown on the right. (**B**) Western blot analysis of PKA, PI3K, P-PI3K, AKT, P-AKT, CaMKII, and β-actin in HAECs infected with Ad-sh-GPR35 and Ad-β-gal. n = 6 per group. Representative bands are shown on the right. (**C**) Western blot assay of eNOS and caveolin-1 expression in HAECs infected with Ad-sh-GPR35 or Ad-*β*-gal. HAEC lysate was loaded and immunoblotted with antibodies against human eNOS, caveolin-1, and *β*-actin (loading control). Quantitative analysis of the expression level of caveolin-1 in HAECs lysates. n =4 per group. (**D**) Western blot assay of eNOS-caveolin-1 association in HAECs with Ad-shGPR35 or Ad-*β*-gal infection. HAECs lysates (300μg) were immunoprecipitated with anti-eNOS and immunoblotted with anti-caveolin-1 and anti-eNOS. N = 4. p > 0.05. The p values were determined by the Mann-Whitney U test between the two groups. In all the dot-plot figures, horizontal lines show mean ± SD. 

**Figure S3**

**Figure S3. GCH1 expression at mRNA levels in endothelial cells.** (**A**) Expression of GCH1 in GPR35^WT^ and GPR35^KO^ MAECs by qPCR. n = 5 per group. (**B**) Expression of GCH1 in HAECs infected with Ad-shGPR35 using Ad-β-gal as control by qPCR. n = 6 per group. p > 0.05. The p values were determined by the Mann-Whitney U test. In all the dot-plot figures, horizontal lines show mean ± SD. 

 

**Figure S4**

**Figure S4. Heart rate in GPR35**^**WT**^ **and GPR35**^**KO**^ **mice under physiological condition.** The heart rate of GPR35^WT^ and GPR35^KO^ adult male and female mice by tail-cuff method. p > 0.05 between GPR35^WT^-female and GPR35^KO^-female. One-way ANOVA was used across all the groups, and if significant, pairs of focused groups were tested with two sample *t*-test and p values were adjusted with Hommel’s method. In all the dot-plot figures, horizontal lines show mean ± SD. 

**Figure S5**

**Figure S5.** **Subunit expression of NADPH oxidase in GPR35**^**WT**^ **and GPR35**^**KO**^ **MAECs.** Western blot analysis of protein expression of Nox2 (**A**), Nox4 (**B**), p22^phox^ (**C**), p47^phox^ (**D**) and β-actin in GPR35^WT^ and GPR35^KO^ MAECs. Representative bands are shown underneath the dot-plots. n = 5 per group, ‘*’ denotes p < 0.05, *P* values were determined by the Mann-Whitney U tests. In all the dot-plot figures, horizontal lines show mean ± SD.

**Figure S6**

**Figure S6. Cell functions of GPR35 MAEC treated with Angiotensin II (Ang II).** MAECs were isolated from GPR35^WT^ and GPR35^KO^ mice, received Ang II treatment, using PBS as control. Cell functions were evaluated by functional assays. (**A**) 2-D network formation. n = 4 per group, Kruskal-Wallis tests were performed across all the groups, and if significant, the pairs of our primary interest were tested using the Mann-Whitney U test with Hommel’s adjustment for multiple comparisons.  ‘*’ denotes p < 0.05. Representative images are shown below. Scale bar = 500 µm). (**B**) Cell proliferation assay.  n = 4 in GPR35^WT^ groups and n = 3 in GPR35^KO^ groups. (**C**) Cell migration by scratch assay**.** n = 4 in GPR35^WT^ groups and n = 3 in GPR35^KO^ groups. Representative pictures are shown below. Scale bar = 200 µm). In all the dot-plot figures, horizontal lines show mean ± SD.

**Figure S7**

**Figure S7. Cell functions and levels of phosphorylated eNOS in mouse aortic endothelial cells from GPR35 male and female mice.** (**A**) Cell migration of MAECs from male and female GPR35^WT^ and GPR35^KO^ mice. n = 4 per group. ‘*’ denotes p < 0.05 vs. Male-WT. Representative pictures are shown next to the dot-plots. Scale bar = 200 µm.  (**B**) Cell proliferation of MAECs isolated from male and female GPR35^WT^ and GPR35^KO^ mice. ‘*’ denotes p < 0.05 vs. Male-WT. (**C**) Levels of phosphorylated eNOS protein in MAECs isolated from male and female GPR35^WT^ and GPR35^KO^ mice. In all the figures, Kruskal-Wallis tests were performed across all the groups, and if significant, the pairs of our primary interest were tested using the Mann-Whitney U test with Hommel’s adjustment for multiple comparisons.  Horizontal lines show mean ± SD..
