# Supplementary material for: Novel Role of GPR35 (G-Protein–Coupled Receptor 35) in the Regulation of Endothelial Cell Function and Blood Pressure
Source: Hypertension. 2021 Jul 19;78(3):816–30. doi: 10.1161/HYPERTENSIONAHA.120.15423 (PMC8357038; doi:10.1161/HYPERTENSIONAHA.120.15423)
Supplement: Supplementary file 1 [file hyp-78-816-s003.pdf]

*** Short In Vivo Checklist**
AHA - Preclinical Animal Testing: Prevention of bias is important for experimental cardiovascular research. ***This short checklist must be completed, and the answers should be clearly presented in the manuscript as well.*** *The checklist will be used by reviewers and editors but will not be published. If a revision is requested, you will be required to complete at revision submission a more detailed checklist that will be published with the accepted article.*

This study involves animals:
Yes

Animals

Species, age, sex, strains, and sources of animals are described: Yes

Randomization

Randomization and allocation concealment were performed: Yes

Blinding

Blinding was performed: Yes

Inclusions and Exclusions (a)

Specific criteria for inclusions and exclusions are specified: Yes

Inclusions and Exclusions (b)

Criteria for inclusions and exclusions were set before the study: Yes

Reporting of Excluded Animals

All animals excluded after the randomization are reported: Yes

Statistical Methods

Statistical Methods are described: Yes

Date completed: 06/09/2021 15:09:29
User pid: 434708
